# Imaging of Focal Autoimmune Pancreatitis and Differentiating It from Pancreatic Cancer

**DOI:** 10.5402/2013/569489

**Published:** 2013-01-16

**Authors:** Abhishek Vijayakumar, Avinash Vijayakumar

**Affiliations:** ^1^Department of General Surgery, Victoria Hospital, Bangalore Medical College and Research Institute, 128 Vijay Doctors Colony, Konanakunte, Bangalore, Karnataka 560062, India; ^2^Department of Radiology, Banaras Hindu University, Varanasi, Uttar Pradesh 221005, India

## Abstract

Autoimmune pancreatitis (AIP) is an inflammatory disorder of pancreas. Two types have been identified: the diffuse and the focal or mass forming. Clinical presentation of AIP overlaps that of pancreatic cancer (PC). Sometimes serum IgG4 and CA 19-9 levels are unable to differentiate AIP from PC. Various series have shown that 5%–21% of resected pancreatic masses for suspected malignancy turned out to be AIP. Accurate diagnosis of focal AIP can avoid unnecessary surgeries. This paper elaborates the various imaging modalities useful in differentiating focal AIP from PC.

## 1. Introduction

Autoimmune pancreatitis (AIP) is now a well-defined entity among the inflammatory diseases of the pancreas [1]. According to the Asian Diagnostic Criteria for Autoimmune pancreatitis [2], AIP has been classified into diffuse and focal types. Focal AIP is characterized by a segmental involvement of the parenchyma with the possibility of a low-density mass being present at imaging. Clinically, AIP patients and patients with pancreatic cancer (PC) share many features, such as preponderance of elderly males, frequent initial symptom of painless jaundice, development of new-onset diabetes mellitus, and elevated levels of serum tumor markers. Radiologically, focal swelling of the pancreas, the “double-duct sign,” representing strictures in both biliary and pancreatic ducts, and encasement of peripancreatic arteries and portal veins are sometimes detected in both AIP and PC. Several series indicate that in 5%–21% of resected pancreatic masses suspected of being cancerous, the final diagnosis was AIP [3–5].

Since AIP responds dramatically to steroid treatment, a correct diagnosis of the disease is important to avoid surgery. On the other hand, in the presence of a resectable pancreatic mass, the probability of cancer is very high (>90%). A misdiagnosis of AIP implies 2-3 weeks of steroid treatment and a one month delay in surgery, with the consequent risk of not operating because of the progression of the malignancy with the onset of metastasis or of vascular involvement.

Korean Society of Gastroenterology proposed new diagnostic criteria to diagnose AIP (Table 1) which included imaging finding, serology, histopathology, and response to steroid treatment [6].

There are some diagnostic difficulty in differentiating focal AIP and PC. Though serum IgG4 levels are elevated in AIP it was observed in a study by Tabata et al. [7], that only 77% of the 39 AIP cases had IgG4 levels > 135 mg/dL. It was also observed that 5 of the 114 cases of PC had IgG4 > 135 mg/dL. Also, when the cutoff of IgG4 was raised to 280 mg/dL, only 1% of PC showed elevated IgG4, compared to 53% of AIP cases [8]. Thus, serum levels of IgG4 alone cannot be used to differentiate AIP from PC.

Tumor markers have been evaluated to differentiate benign from malignant pancreatic masses, and one such marker is CA 19-9. In a study by Marrelli et al. [9], it was found that when a cutoff of 90 U/mL was used, the sensitivity and specificity were 85% and 87%, respectively, to differentiate benign from malignant lesions. Further Bedi et al. [10] showed that raising the cutoff of CA19-9 to 1000 U/mL had specificity of 99.8%, but sensitivity was only 41%. Chari et al. [11], retrospectively, compared 48 patients with autoimmune pancreatitis presenting with obstructive jaundice and 100 patients with pancreatic cancer. Serum CA 19-9 of more than 150 U/mL was more than 90% specific for pancreatic cancer. CA 19-9 lacks sensitivity for early or small-diameter pancreatic cancers, and only 50% of patients with pancreatic cancers <3 cm were found to have elevated levels of CA 19-9 [12]. Thus, tumor marker cannot accurately differentiate AIP from PC. 

## 2. Dual Phase CT

Computer tomography (CT) imaging is an essential criterion in diagnosis of AIP. The characteristic CT appearance of autoimmune pancreatitis has been described as diffuse enlargement of the pancreas with a capsule-like rim. The pancreatic border becomes featureless with effacement of the lobular contour of the pancreas. There is diffusely decreased enhancement in the pancreas during the early phase and delayed enhancement in the late phase of contrast enhancement. Involvement of other organs, such as the biliary tree, retroperitoneum, salivary glands, and kidneys, is common [13]. In focal AIP with mass formation, it is necessary to differentiate pancreatic cancer. Dual phase CT has been used to diagnose AIP based on enhancement pattern of pancreas. Yang et al. [14] described the enhancement of the pancreas in 20 patients with autoimmune pancreatitis imaged with dual-phase CT technique. The CT attenuation of the pancreas in autoimmune pancreatitis was similar to or greater than that of the liver in both the pancreatic and the hepatic phases. Wakabayashi et al. [15] evaluated the CT enhancement pattern of a focal form of autoimmune pancreatitis in nine patients. In the nine patients, six lesions were hypoattenuating in the early phase, but all were homogeneously isoattenuating in the delayed phase. Only two of 80 malignant tumors had homogeneous enhancement in the delayed phase.

In a study by Takahashi et al. [16], delayed enhancement of the mass, or focally enlarged segment, was defined as a 15-HU or greater increase from the pancreatic phase to the hepatic phase. In autoimmune pancreatitis, the pancreas had decreased enhancement in the pancreatic phase of CT and nearly normal enhancement in the hepatic phase. In comparison, in normal pancreases, the parenchyma had maximum enhancement in the pancreatic phase and washout in the hepatic phase. Pancreatic carcinoma had decreased enhancement in the pancreatic phase and minimal change in enhancement in the hepatic phase. This finding may help differentiate the focal form of autoimmune pancreatitis from pancreatic carcinoma, although acute pancreatitis complicating pancreatic carcinoma may have a similar enhancement pattern in the tumor-free segments.

Main pancreatic duct stenosis is found frequently in PC and AIP in a study by Kawai et al. [17]. It was shown that MPD enhancement was found in 67% of the cases of AIP compared to 10% of PC and none of chronic pancreatitis cases. Also, there was enhancement of duct downstream of the mass. Thus, duct enhancement sign can be used to differentiate AIP from PC.

## 3. MRI

MRI is a useful tool to evaluate pancreatic lesions. Contrast-enhanced fat suppression MRI has been studied to differentiate AIP and PC. In a study by Sugiyama et al. [18], it was shown that speckled enhancement within a hypointense or isointense lesion on pancreatic phase DCE-T1WI (speckled type) was observed more frequently in focal AIP than in PC, with high sensitivity, high specificity, and high accuracy. Hypointensity to hyperintensity surrounding a less enhanced focal area on DCE-T1WIs (target type) and upper stream main pancreatic duct dilatation were observed more frequently in PC than in f-AIP. Speckled enhancement inside a focal AIP lesion on pancreatic phase DCE-T1WI is useful for differentiation from PC.

Diffusion weighted MRI (DW-MRI) has been used to differentiate AIP from PC. Both AIP and PC were detected as high signal intensity areas. However, the high signal intensity areas were found to be diffuse, solitary, and multiple in AIP patients, whereas all patients with PC had solitary areas. Additionally, the apparent diffusion coefficient (ADC) values were significantly lower in AIP than in PC patients or in individuals with a normal pancreas. Morphological differences seen in high signal intensity areas on DW-MRI and ADC values may prove useful to help distinguish AIP from PC [19].

## 4. FDG PET

The role of ^18^F-FDG PET in diagnosis of AIP from other chronic pancreatitis was described by Nakamoto et al. [20]. Though AIP like other chronic pancreatitis is predominated by fibrosis, the active inflammation with dense lymphoplasmacytic cells infiltrate causes increased FDG uptake. In a study by Tae et al. [21] comparing PET CT features of focal AIP and PC, it was seen that 53% (*n* = 17) of AIP cases showed diffuse uptake of FDG in comparison to 3% (*n* = 151) of pancreatic cancer. The diffuse uptake in cases of pancreatic cancer was due to obstructive pancreatitis which could be differentiated by other CT features. In a study by Ozaki et al. [22], heterogeneous FDG accumulation was found in almost all cases (14 of 15) of AIP, and homogeneous accumulation was frequently seen in patients with pancreatic cancer. Though extrapancreatic FDG uptake was found in both AIP and pancreatic cancers, the increased uptake in salivary gland and kidney was specific to AIP. Also, PET CT can be used to monitor response to steroid treatment with decrease in uptake in pancreas and extra pancreatic sites [21]. The presence of diffuse pancreatic uptake or concomitant extrapancreatic uptake by the salivary glands at PET/CT may be used to aid in differentiation of autoimmune pancreatitis from pancreatic cancer in difficult cases.

## 5. ERCP and MRCP

Pancreatographic demonstration of narrowing of main pancreatic duct (MPD) forms essential criteria in diagnosis of AIP. In a study by Kamisawa et al. [23], obstruction of the MPD was detected more often in PC patients (60%) than in AIP patients (6%). The length of the narrowed portion of the MPD on endoscopic retrograde pancreatography (ERP) was 6.7 ± 3.2 (mean ± SD) cm in AIP patients, which was significantly longer than in PC patients (2.6 ± 0.8 cm ). The length of the narrowed portion of the MPD on ERP was longer than 3 cm in 76% of AIP patients as compared to 20% of PC. In AIP patients, the degree of narrowing of the MPD varied in the same patient, and skipped, narrowed lesions of the MPD were detected in 35% of our AIP patients, but in none of the PC patients. In AIP patients with segmental narrowing of the MPD, upstream dilatation of the distal MPD was less often noted than in PC. The maximal diameter of the upstream MPD on ERP was 2.9 ± 0.7 mm in segmental AIP patients, which was significantly smaller than in pancreatic head cancer patients (7.1 ± 1.9 mm). The maximal diameter of the upstream MPD was smaller than 5 mm in 94% of segmental AIP patients. Side branches were more frequently derived from the narrowed portion of the MPD in AIP patients (65%) than in PC patients (25%). Similar results were seen in studies by Wakabayashi et al. [15] and Takuma et al. [24] comparing ERCP findings in AIP and PC.

Magnetic resonance cholangiopancreatography (MRCP) has become popular as a noninvasive method for obtaining high quality images of the pancreaticobiliary tree; MRCP is replacing diagnostic ERCP in many pancreatobiliary diseases. There has been a controversy in usefulness of MRCP in diagnosis of AIP. The major problem with MRCP for diagnosing AIP is that the narrowed MPD seen on ERCP cannot be visualized on MRCP, because of the inferior resolution of MRCP compared with ERCP. Segmental narrowing of the MPD seen on ERCP was not visualized in 86% on MRCP, and distinguishing between AIP and PC was quite difficult on MRCP. However, in these cases, less upstream dilatation of the MPD on MRCP may suggest AIP rather than PC [25]. With newer MRCP models and secretin, MRCP spatial resolution can be increased and could emerge as accurate imaging modality to diagnose and differentiate AIP and PC.

Stenosis of lower bile duct is common to both AIP and PC. ERC findings in patients with AIP may help differentiate this condition from common bile duct (CBD) cancer. Although it is found that smooth margins, gradual and symmetric narrowing, and fully visible lumen were more common in AIP, they were not specific to AIP as they are also seen in 22%–58% of patients with CBD cancer. However, an hourglass appearance is highly specific for AIP as it was observed in only 9% (8/93) of patients with CBD cancer. Strictures confined to the intrapancreatic CBD at CT and showing an hourglass appearance at ERC would therefore be characteristic of AIP [26]. Other supportive features for diagnosis of AIP are long segment strictures and occasional involvement of intrahepatic bile ducts. Though the intrahepatic stricture can look similar to primary sclerosing cholangiopathy, the diffusely distributed, beaded, and pruned-tree appearance that is usually detected in PSC patients is not detected in AIP patients. Stenosis of the hilar bile duct in AIP patients should be also differentiated from cholangiocarcinoma at the hepatic hilus these diseases can be differentiated by the absence of the pancreatic abnormalities in patients with cholangiocarcinoma at the hepatic hilus [27].

## 6. Endoscopic Ultrasound (EUS)

Endoscopic ultrasonography (EUS) is superior to standard imaging techniques in detecting pancreatic cancer or masses and in the assessment of early parenchymal changes in chronic pancreatitis [28, 29]. Endoscopic ultrasonography (EUS) imaging of AIP shows hypoechoic enlargement of the pancreas with hypoechoic spots. A lobular outer gland margin of the pancreas or a hyperechoic pancreatic ductal margin, which is frequently detected in alcoholic chronic pancreatitis, is rarely observed in AIP patients. In the focal form of AIP, a solitary, irregular hypoechoic mass, generally located in the head of the pancreas, is observed [30, 31]. Hyperechoic spots in a hypoechoic mass and the duct-penetrating sign suggest AIP rather than PC. Hyperechoic spots may correspond to compressed pancreatic ducts [32]. 

Administration of contrast agents is another way to improve EUS-based diagnosis of solid pancreatic tumors. Modern contrast enhanced EUS relies on a dedicated contrast harmonic echo (CHE-EUS) technique that detects signals from microbubbles delivered by new contrast agents like Sonovue in vessels with very slow flow without the burden of Doppler-related artifacts. Fusaroli et al. [33] investigated 90 patients with solid pancreatic lesions by CEH-EUS, using Sonovue as contrast agent. The finding of a hypoenhancing mass with an inhomogeneous pattern diagnosed pancreatic adenocarcinoma with a sensitivity of 96% and an accuracy of 82%. The study also indicated that this CEH-EUS pattern diagnosed malignancy more accurately than the finding of a hypoechoic mass on standard EUS. Hyperenhancement specifically excluded adenocarcinoma (98%), although sensitivity was low (39%). 

Seicean et al. [34] investigated the possibility to use quantitative CEH-EUS data in the differential diagnosis between pancreatic cancer and chronic pancreatitis. A hypoenhanced pattern was the most common finding both in with pancreatic adenocarcinoma (14/15 cases) and in mass forming chronic pancreatitis (10/12 cases). However, an index of contrast uptake ratio was calculated, and this was significantly lower in adenocarcinoma compared to cases with mass-forming chronic pancreatitis. A cutoff uptake ratio index value of 0.17 for diagnosing adenocarcinoma corresponded to a sensitivity of 80%, a specificity of 91.7%, a positive predictive value of 92.8%, and a negative predictive value of 78%. Generally, differences in histology, such as histological differentiation grade, amount of fibrosis, and obliteration of blood vessels in the tumor, may be associated with differences in enhancement behavior. 

In cases where it is difficult to differentiate a hypoechoic mass as AIP or PC, EUS elastography can be a useful tool. EUS elastography is a noninvasive technique that measures elasticity in real time by registration of differences in distortion of the EUS image after application of slight pressure by the EUS probe. Many different pathological processes, including inflammation, fibrosis, and cancer, can alter tissue elasticity that will result in distinct elastographic appearance. Initial studies by Giovannini et al. [35] were scoring system based on different color patterns in EUS elastography images. Sensitivity and specificity of 100% and 67%, respectively, were observed in the differentiation between benign and malignant pancreatic masses. In a case-control study of five patients with AIP, EUS elastography showed a typical and homogeneous stiffness pattern of the focal lesions and of the surrounding parenchyma that is different from that observed in ductal adenocarcinoma [36]. 

However, substantially lower diagnostic performance by qualitative elastography has been reported. Recently, quantitative EUS elastography has been developed in an attempt to make the elastography interpretation less subjective. Quantitative elastography renders a numeric result, either as mean value of hues in a selected area (mean hue histogram) or as a ratio of elasticity in the target area over soft reference tissue (strain ratio). IglesiasGarcia et al. [37] studied 86 pancreatic masses using quantitative EUS elastography. The strain ratio was significantly higher among patients with malignant pancreatic tumors compared to those with inflammatory masses. Normal pancreatic tissue showed a mean strain ratio of 1.68 (95% CI: 1.59–1.78). Inflammatory masses presented a strain ratio (mean 3.28; 95% CI: 2.61–3.96) significantly higher than that of the normal pancreas but lower than that of pancreatic adenocarcinoma (mean 18.12; 95% CI: 16.03–20.21). The highest strain ratio was found among endocrine tumors (mean 52.34; 95% CI: 33.96–70.71). The sensitivity and specificity of the strain ratio for detecting pancreatic malignancies using a cutoff value of 6.04 were 100% and 92.9%. 

Săftoiu et al. [38] evaluated the usefulness of the hue histograms in a similar setting. Based on a cutoff of 175 for the mean hue histogram value, the sensitivity, specificity, and accuracy of differentiation of benign and malignant masses were 91.4%, 87.9%, and 89.7%, respectively.

EUS allows visualization of the entire common bile duct and enables identification of the cause of a biliary stricture. In patients with either diffuse or focal AIP, EUS can show dilatation of the common bile duct and thickening of its wall better than other diagnostic techniques [39].The typical EUS feature of the common bile duct is a homogeneous, regular thickening of the bile duct wall, called “sandwich-pattern,” which is characterized by an echo-poor intermediate layer and hyperechoic outer and inner layers [40]. A further application of EUS is intraductal ultrasonography (IDUS), which can be performed during endoscopic retrograde cholangiography for the characterization of biliary stenosis. Naitoh et al. [41] recently evaluated IDUS findings in 23 patients with AIP. They found that a circular, symmetric wall thickness, smooth inner and outer margins, and a homogeneous intermediate layer in the stricture were significantly more common in AIP than in cholangiocarcinoma. 

Other EUS features of AIP include peripancreatic lymphadenopathy and vascular invasion which are very similar to PC. EUS can detect single or multiple enlarged lymph nodes in patients with AIP, reflecting the underlying inflammatory process, which can involve extra-pancreatic organs [42]. Hoki et al. [43] reported a significant difference in detection of lymphadenopathy by EUS imaging over CT (72% versus 8%) in patients with AIP. Moreover, in the same series, a trend toward a higher prevalence of lymphadenopathy in AIP compared to pancreatic cancer was reported. The typical features of metastatic nodes on EUS like size >1 cm, hypoechoic appearance, round shape, and smooth borders are not very accurate in pancreaticobiliary cancers [44].

Retroperitoneal fibrosis and sclerosing mesenteritis are common features of AIP resulting in narrowing of mesenteric vessels. In a series of 14 patients with AIP, EUS suspected invasion of the portal or mesenteric veins in 21% of patients compared to 14% on CT. No pancreatic cancer developed during the followup of these patients. Such EUS features, easily mistaken for malignancy, are due to the inflammatory process of AIP, which can involve medium and large-sized vessels [45]. 

## 7. Endoscopic Fine Needle Aspiration Cytology and Biopsy (EUS-FNA, EUS-TCB)

According to clinical diagnostic criteria of AIP, histological presence of fibrosis and lymphoplasmacytic infiltration of pancreas is diagnostic of AIP. In cases of diffuse AIP with typical imaging features and raised serum IgG4, it is not necessary to perform a biopsy, a response to steroid is enough to prove the diagnosis of AIP. In cases of focal or mass forming AIP, it is necessary to exclude pancreatic carcinoma before starting steroid treatment. EUS-FNA is an established and widely used technique to evaluate pancreatic masses. The diagnostic accuracy of EUS-FNA for pancreatic cancer is reported to be between 60% and 90% [46, 47]. Fritscher-Ravens et al. [48] found that sensitivity of EUS-FNA in patients with a focal pancreatic lesion without chronic pancreatitis was 89%, while it was only 54% in patients with chronic pancreatitis. 

EUS-FNA can be employed to yield specimens of pancreatic lesions, the common bile duct wall, or lymph nodes. Although a cytologic pattern specific for AIP has not been identified, high cellularity of stromal fragments with lymphoplasmacytic infiltrate has emerged as a discriminating feature. In a study by Deshpande et al., 56% of AIP patients presented such a feature versus 19% of patients with pancreatic carcinoma, and none of the chronic pancreatitis controls exhibited this feature [49]. 

Sometimes histological diagnosis of AIP is often difficult due to the small size of specimens obtained by FNA. Recently, there have been several reports on the usefulness of EUS-guided trucut biopsy (EUS-TCB) for the diagnosis of AIP [50, 51]. Trucut biopsy needles have been developed to acquire samples while preserving tissue architecture, thus allowing histological examination. Previous reports describe the safety and the technical feasibility of performing EUS-TCB from a transgastric approach [52]. However, the TCB device may not function properly when used in the second portion of the duodenum, and there is also some difficulty when using the TCB device from the duodenal bulb and along the greater curvature of the antrum. In a study by Mizuno et al. [53], they compared EUS-FNA and EUS-TCB performed in 14 patients for the diagnosis of AIP. EUS-TCB showed higher sensitivity (100%) and specificity (100%) compared to EUS-FNA (36% and 33%, resp.). 

With difficulty in obtaining adequate specimen and complications associated with EUS-FNA and EUS-TCB, there have been studies evaluating duodenal papillae biopsy as an alternative to pancreatic tissue in diagnosis of AIP. Compared with the pancreatic biopsy material, the specimens taken from the duodenal papilla can be retrieved in an easy, safe, and reliable way [54]. Kamisawa and colleagues [55] revealed that the results of endoscopic biopsy using IgG4 immunostaining were useful in the differential diagnosis between patients with AIP and PC. Abundant infiltration of IgG4-positive plasma cells in the papilla is frequently and specifically detected in AIP patients. In their study of IgG4 immunostaining in biopsy specimens from the major duodenal papilla, severe infiltration of IgG4-positive plasma cells (≥10/HPF (high power field)) was observed in the major duodenal papilla of all 8 AIP patients with pancreatic head involvement. Moderate infiltration of IgG4-positive plasma cells (9–4/HPF) was detected in 1 patient with pancreatic head cancer. IgG4 immunostaining of biopsy specimens obtained from the major duodenal papilla is useful for supporting a diagnosis of AIP with pancreatic head involvement.

## 8. Conclusions

Autoimmune pancreatitis is a form of chronic pancreatitis with clinical features overlapping that of cancer. Focal autoimmune pancreatitis needs to be differentiated from pancreatic cancer before starting steroid therapy. With cases where both serum IgG4 and CA 19-9 levels are nondiagnostic of AIP or PC, imaging finding can help in diagnosis of AIP and avoid unnecessary surgery. Dual phase CT, MRI, FDG PET, MRCP and EUS elastography are noninvasive ways with which AIP can be differentiated from PC (Table 2). ERCP, EUS-FNA, and EUS-TCB can be reserved in cases of atypical imaging findings to diagnose AIP and PC.

## Figures and Tables

**Table 1:** Diagnostic criteria for autoimmune pancreatitis proposed by the Korean Society of Gastroenterology.

Criterion I. Imaging (both of the following required)	
(1) Imaging (CT or MRI) of pancreatic parenchyma: diffusely/segmentally/focally enlarged gland, occasionally with mass and/or hypoattenuation rim.	
(2) Imaging (ERCP or MRCP) of pancreaticobiliary ducts: diffuse/segmental/focal pancreatic ductal narrowing, often with stenosis of bile duct.	
Criterion II. Serology (one of the following required)	
(1) Elevated levels of serum IgG (>1800 mg/dL) or IgG4 (>135 mg/dL)	
(2) Detected autoantibodies.	
Criterion III. Histopathology of pancreatic/extrapancreatic lesions (one of the following required)	
(1) Lymphoplasmacytic infiltration and fibrosis, often with obliterative phlebitis.	
(2) Presence of abundant (>10 cells/high power field) IgG4-positive plasma cells.	
Criterion IV. Response to steroids	
Resolution/marked improvement of pancreatic/extrapancreatic lesion with steroid therapy.	
Probable diagnosis: Criterion V or VI	
Criterion V. Unexplained pancreatic disease but only with characteristic pancreatic histology.	
Criterion VI. (Both of the following required)	
(1) Other organ involvement and/or serologic abnormalities.	
(2) Various atypical pancreatic imaging suggesting chronic pancreatitis with negative workup for known etiologies	

Adapted from Kim and Lee [6].

CT: computed tomography; ERCP: endoscopic retrograde cholangiopancreatography; IG: immunoglobulin; MRCP: magnetic resonance cholangiopancreatography; MRI: magnetic resonance imaging.

**Table 2:** Imaging features of focal AIP and PC.

Investigation	Focal AIP	Pancreatic cancer
Dual phase CT	Decreased enhancement in pancreatic phase, normal or delayed enhancement in hepatic phase. Enhanced duct sign	Decreased enhancement in pancreatic phase, decreased or minimal increase in enhancement in hepatic phase.

MRI	Speckled appearance within hypointense lesion.	Target-like lesion with upstream dilatation of MPD.
	Low diffusion coefficient on DW-MRI	High diffusion coefficient on DW-MRI.

^ 18^FDG-PET CT	Diffuse FDG uptake	Focal FDG uptake.
	Uptake in salivary gland and kidney	

ERP/pancreatography	Long segment narrowing of MPD > 3 cm, skip lesions, upstream dilatation of MPD < 5 mm, side branch dilatation from narrowed MPD.	Complete MPD obstruction, short segment narrowing <3 cm, upstream dilatation of MPD > 5 mm.

ERC/cholangiography	Lower bile duct stenosis smooth margins, gradual and symmetric narrowing, and fully visible lumen or hourglass appearance.	Short segment stenosis irregular margins, complete obstruction.
	Intrahepatic biliary stricture.	

EUS	Hyperechoic spots in a hypoechoic mass and the duct-penetrating sign.	Hypoechoic mass with inhomogeneous pattern. Low contrast uptake index on CHE-EUS.
	Delayed enhancement in CHE-EUS.	
	Increased thickness of CBD with “sandwich pattern”	
	Peripancreatic lymphadenopathy	

EUS elastography	Strain ratio <4, hue histogram value <175	High strain ratio >18, hue histogram value >175.

EUS-FNA, EUS-TCB	High stromal cellularity with lymphoplasmacytic infiltrates.	Features of carcinoma.
	High immunochemical staining with IgG4.	

CT: computed tomography; MRI: magnetic resonance imaging; DW-MRI: diffusion weighted magnetic resonance imaging; MPD: main pancreatic duct; CBD: common bile duct; FDG PET: 18 fluro deoxyglucose positron emission tomography; EUS: endoscopic ultrasound; CHE-EUS: contrast harmonic echo endoscopic ultrasound; EUS-FNA: endoscopic fine needle aspiration cytology; EUS-TCB: endoscopic trucut biopsy.
